# Salient semantics

**DOI:** 10.1007/s11229-024-04669-x

**Published:** 2024-07-17

**Authors:** Kevin Reuter

**Affiliations:** Institute of Philosophy, University of Zurich, Zürichbergstrasse 43, 8044 Zurich, Switzerland

**Keywords:** Salience, Semantic features of concepts, Semantic feature production tasks, Necessity, Conceptual analysis, Conceptual engineering, Theories of concepts

## Abstract

Semantic features are components of concepts. In philosophy, there is a predominant focus on those features that are necessary (and jointly sufficient) for the application of a concept. Consequently, the method of cases has been the paradigm tool among philosophers, including experimental philosophers. However, whether a feature is salient is often far more important for cognitive processes like memory, categorization, recognition and even decision-making than whether it is necessary. The primary objective of this paper is to emphasize the significance of researching salient features of concepts. I thereby advocate the use of semantic feature production tasks, which not only enable researchers to determine whether a feature is salient, but also provide a complementary method for studying ordinary language use. I will discuss empirical data on three concepts, conspiracy theory, female/male professor, and life, to illustrate that semantic feature production tasks can help philosophers (a) identify those salient features that play a central role in our reasoning about and with concepts, (b) examine socially relevant stereotypes, and (c) investigate the structure of concepts.

## Theoretical background

### What are salient features?

According to most psychological and philosophical theories of concepts, the majority of concepts can be analyzed into sets of features or components. Those features have different attributes.^1^ Advocates of classical theories of concepts are mostly interested in whether a feature has the attribute of *necessity*, i.e., whether the feature is a necessary component of a concept. Advocates of prototype and exemplar theories care more about whether a feature is *typical* or prototypical, i.e., whether most objects that fall under a concept have the property referred to by that feature, or whether a particularly noteworthy exemplar has that feature.

Necessity and typicality are not the only attributes that play an important role in our representation of kinds. A feature might be *universal*, i.e., all objects falling under a concept have the property referred to by that feature, but not necessarily so. A feature might also be *salient*, which means—very roughly—that some objects falling under a concept have a property referred to by that feature that is striking, i.e., it stands out from other properties in our representation of the kind.^2^

Here is an example to illustrate differences between those attributes. The concept of shark might be characterized to have the following set of features: <is a fish>, <has 5–7 pairs of gills>, <is predatory>, <attacks humans>. Sharks are necessarily fish. <is a fish> is thus a necessary feature of shark. All sharks have 5–7 pairs of gills, but sharks might evolve to have more than 7 pairs of gills. The feature <has 5–7 pairs of gills> is thus universal without being necessary. Most sharks are predatory, but not all are. <is predatory> is thus only a typical, but not a universal or even necessary feature of shark. And hardly any sharks attack humans, but <attacks humans> is a highly salient feature, most likely because the potential danger of sharks plays an important role in people’s reasoning about sharks.

Although I have provided a tentative characterization of salience above and have discussed an example of a salient feature of a concept, readers might still be unclear about what exactly is meant by salience. Unfortunately, there is no accepted definition of salience. Instead, researchers seem to either

- provide paraphrases that are likely to be uninformative and possibly circular (Sloman et al., 1998, my own characterization above);
- state technical definitions (Del Pinal & Spaulding, 2018; Fischer & Engelhardt, 2020; Sloman et al., 1998);
- operationalize instead of provide a definition (McRae et al., 2005).

Let me quickly take these three approaches in turn. First, stating that features are salient if and only if the property they refer to is striking (see my characterization above) seems to only trade one word for a synonymous term. More worryingly, if one is pressed to say what it is for a property to be striking, one is easily led into a circle by saying that a striking property is salient in our representation of the kind. Sloman et al.’s (1998) claim that salient features are those that are ‘prominent’ in our representation does not fare any better, unless an informative definition of ‘prominent’ is given, which is at least absent in Sloman et al.’s discussion.

Second, Sloman et al. also provide a more technical definition of salience:

Salience refers to the intensity of a feature, the extent to which it presents a high amplitude signal in relation to background noise, in a way that is fairly independent of context. For example, the brightness of a bright light or the redness of a fire engine are salient features. (1998, p. 193)

It seems that Sloman et al. take the analogy with perceptual salience quite literally. However, while perceptual salience of a stimulus and the salience of a feature of a concept might share some commonalities, the analogy breaks down fairly quickly when we consider functional and abstract features. For example, it is hard to tell what the signal-to-noise ratio for the feature <attacks humans> is supposed to be, or how to cash out the salience of features for abstract concepts like conspiracy theory or life in terms of its intensity or high amplitude signal. In a range of projects, Fischer and Engelhardt investigate reasoning processes that are influenced by what they call a linguistic salience bias. While their focus is more strongly on stereotypical inferences due to dominant uses of words (see e.g., Fischer & Engelhardt, 2016, Fischer & Engelhardt, 2019, see also Fischer & Sytsma, 2021), they also provide an extended characterization of salience:

Salience (in this sense) is a function of exposure frequency, that is, of how often the language user encounters the word in this sense. It is further modulated by prototypicality (Rosch, 1978), where a sense of a polysemous word (e.g., “see”) is more or less prototypical depending upon whether it stands for more or less prototypical examples of the relevant category (e.g., more or less prototypical cases of seeing). The more salient a use is for a hearer, the more rapidly and strongly the situation schema associated with it gets activated. (Fischer & Engelhardt, 2020, p. 418)

Cashing out salience as a function of frequency and prototypicality seems very promising, especially given the results from empirical studies (see below). A possible downside of this characterization is that prototypicality is usually considered to be itself a function of frequency, and thus, we would need to know more about Fischer & Engelhardt’s definition of prototypicality. Additionally, their work focuses mostly on polysemous uses of terms, for which we can expect other factors to play a more important role for salience.

Other researchers (e.g., McRae et al., 2005) take the salience of a feature to be what is revealed by certain experimental tasks, e.g., memory retrieval tasks. However, this approach rather operationalizes the concept of salience but does not provide an independent definition. Consequently, we cannot tell whether a feature is salient because it is quickly retrieved from memory or whether a feature is quickly retrieved from memory because it is salient. With this not entirely satisfying state of affairs, let us consider the role that salience plays for traditional philosophers, experimental philosophers and psychologists.

### (Experimental) Philosophers and psychologists

In the practice of conceptual analysis, philosophers typically focus on identifying components that are necessary for applying a concept. This approach often leads them to adopt the classical theory of concepts. According to this theory, concepts are understood as collections of features that are both necessary and jointly sufficient for their application.^3^,^4^ The dominant method of conceptual analysis for determining the necessary and jointly sufficient features of a concept is the method of cases: Philosophers devise thought experiments that constitute possible or actual counterexamples to the proposed definition of a concept.

Experimental philosophers have challenged the idea that a small number of experts, i.e., professional philosophers, can reliably and robustly reveal whether a putative counterexample works or whether it fails (Brun & Reuter, 2022; Mallon et al., 2009). Most experimental philosophers, however, rarely challenge the predominance of the search for necessary and jointly sufficient features of concepts. In other words, the method of cases is also a prevalent approach among experimental philosophers. While vignette studies do not need to be designed to test for necessary and jointly sufficient features, they often are.^5^ Why have philosophers been less intrigued by other attributes of features, especially by the attribute of salience? Arguably, the issue of feature salience has been largely neglected in philosophical debates because philosophers commonly hold that their main objective is to probe deeper than the obvious or ‘salient’ aspects that are immediately accessible to most individuals. Instead, their goal is to venture past these initial impressions to uncover the more intricate and profound essence of various phenomena. However, this paper argues that disregarding the salience of features as irrelevant to philosophical inquiry is a significant oversight. It will demonstrate that acknowledging and examining the salience of features is not just beneficial but essential for a comprehensive and accurate understanding of various concepts, offering a richer and more complete perspective in philosophical explorations.^6^

Several scholars, including Machery (2017) and Isaac (2021a, b), have underscored the importance of adopting psychological approaches in analyzing concepts and highlighting areas for conceptual change. Despite this, the influence of feature salience on our engagement with and understanding of philosophical concepts remains largely uncharted territory. A notable exception can be found in the works of Fischer and his colleagues, who investigate the effects of what they term “linguistic salience bias” on reasoning processes, as seen in Fischer and Engelhardt (2016, 2020), and Fischer and Sytsma (2021). Aside from Fischer and colleagues’ work on the linguistic salience bias, the importance of the salience of a feature of a concept has been noted in the study of generics, see, in particular, Leslie (2008). According to Leslie’s view, if a particular feature (like <attacks humans>) is highly salient in a concept like shark, people are likely to formulate a generic statement based on that feature (“Sharks attack humans”), even if the characteristic is not statistically prevalent in the category of sharks. Despite the significance of feature salience for the study of generics, little empirical work has in fact been conducted to determine the salient features of concepts.

Psychologists, in contrast to both traditional and experimental philosophers, are less impressed by the classical theory of concepts and the search for necessary and jointly sufficient features. Not only are there surprisingly few success stories of the classical theory (Laurence & Margolis, 1999), many studies have shown that our categorization and reasoning processes are often best explained by prototypical representations and encoded exemplars (Rosch, 1978; Hampton, 1995). Whether or not the classical theory can somehow accommodate this research is a matter of ongoing debate (see e.g., Lakoff, 2007; Machery, 2009). I am not taking any sides in this discussion. What can be said though with certainty is that psychologists take very seriously and investigate thoroughly the salience, typicality, centrality, and diagnosticity of features, whereas philosophers consider those attributes of a concept’s features to a much smaller extent.

Despite psychologists’ interest in salient and typical features of concepts, there is relatively little work on the salient and typical features of individual concepts, especially for philosophically relevant concepts. As an example, take the concept of lie for which philosophers and psychologists have advanced our knowledge hand-in-hand. The case of lie underscores my claim that even experimental philosophers are usually strongly invested in the traditional program of finding necessary and jointly sufficient conditions. Coleman and Kay (1981) empirically studied and developed a prototype semantics for the concept lie. Experimental philosophers have contributed widely to the literature on lie within the last 10 years. Although some of this research belongs both philosophically and methodologically to the best of experimental philosophy, those experimental philosophers have, unfortunately in my opinion, ‘gone back’ to frame their results in terms of necessary and sufficient conditions (Rutschmann & Wiegmann, 2017; Turri & Turri, 2015; Wiegmann & Willemsen, 2017).^7^

Thus, on the one hand, we have scholars—the psychologists—who are interested in typical and salient features, but mostly in order to understand how people reason with and about concepts more generally. On the other hand, we have scholars—the philosophers—who are interested in individual concepts like truth, lie, knowledge, conspiracy theory, etc., but then mostly attend to those features of these concepts that might turn out to be necessary for their application.

### The semantic feature production task

The method of cases is not an uncontroversial method, but it surely is the dominant paradigm to explore which features of a concept are considered to be necessary. What about salient features? How can we explore which features are the salient features of a concept? The probably most widely used method to determine salient features of concepts is the so-called semantic feature production task, also known as feature listing task (for some early examples, see e.g., Hampton, 1979; Barsalou, 1983; for an in-depth discussion see, e.g., Machery, 2017).^8^ Interestingly, there is no prescribed way of conducting a semantic feature production task. So, let’s look more closely at how one of the most influential papers (McRae et al., 2005) on semantic feature production tasks goes about doing this (see also Wu & Barsalou, 2009). This is what they presented their participants with:

We want to know how people read words for meaning. Please fill in features of the word that you can think of. Examples of different types of features would be: how it looks, sounds, smells, feels, or tastes; what it is made of; what it is used for; and where it comes from. Here is an example:

duck: is a bird, is an animal, waddles, flies, migrates, lays eggs, quacks, swims, has wings, has a beak, has webbed feet, has feathers, lives in ponds, lives in water, hunted by people, is edible

Complete this questionnaire reasonably quickly, but try to list at least a few properties for each word. (McRae et al., 2005)

After participants were presented with these instructions, they were then simply given a list of words and fields to fill in features that came to mind. These features largely fell into four categories: sensory (e.g., has fur), functional (e.g., you can sit on it), encyclopedic (e.g., lives in woods), and taxonomic (e.g., is a vegetable).

The results of their semantic feature production tasks for 571 items allows for two important observations. First, typicality is arguably the single most important predictor for the frequency with which a feature is named, where typicality is understood as the frequency with which members of a certain category possess the property referred to by the feature.^9^ To illustrate, consider the case of a knife: Not all knifes are sharp, are used for cutting, and have a handle. But many, and perhaps even most, knifes are. Thus, being sharp, used for cutting and having a handle are highly frequent properties of knifes. Thus, rather unsurprisingly, <sharp>, <used for cutting>, <has a handle> are typical features and among the most common features named in a semantic feature production task for knive.

Second, “participants’ responses are somewhat biased toward information that distinguishes among concepts—that is, the pieces of information that enable people to distinguish a concept from other, similar concepts” (2005, p. 549). For example, take the feature <attacks humans> of the concept shark. Sharks hardly ever attack humans. However, other fish are even more unlikely to attack humans. Thus, among all fish, we can distinguish sharks from other fish easily by their propensity to attack humans, even if the propensity is very low. On the flip side, people are very unlikely to state the feature <has a kidney> for the concept human, although all humans have a kidney. <Has a kidney> simply does not allow us to distinguish humans from many other animals. Rosch (1978) made popular the term ‘cue validity’ to refer to the conditional probability of an object falling in a particular category given a particular property. Cue validity is greater the more the feature is considered to apply to members of the category in question, and the less the feature is considered to apply to members of other categories. Thus, <attacks humans> has high cue validity because we associate the property of attacking humans strongly with sharks and with hardly any other fish. And <has a kidney> has relatively low cue validity despite it being universal for humans, because we also associate the property of having a kidney with many other animals.

The more typical and the more cue valid a feature is, the more likely it will be stated frequently in semantic feature production tasks. If we take semantic feature production tasks to reveal some of the most salient features of concepts, then typicality and cue validity seem to be the two most important predictors for salience. Features derived from semantic feature production tasks have been shown to be crucial for cognitive processes like memory, categorization, recognition and even decision-making (Ashcraft, 1978; Cree et al., 1999; Hampton, 1979; Smith et al., 1988; Solomon & Barsalou, 2001; Vigliocco et al., 2004). Thus, the importance of salient features of concepts for various cognitive processes can hardly be overstated.

### Proof of concept

So far, I have introduced the notion of salience (Sect. 1.1). I have then argued that whereas philosophers are interested in individual concepts but rarely in the salient features of those concepts, psychologists are interested in the salient features of concepts, but rarely in any individual concepts that are also relevant for philosophers (Sect. 1.2.). Lastly, I discussed the semantic feature production task as one of the primary methods to reveal the salient features of concepts (Sect. 1.3).

Perhaps, salient features of concepts have not been discussed very much by philosophers, because salient features are simply not particularly interesting when individual concepts are at stake. Thus, the burden of proof is certainly on those like me who argue that we should care about whether a feature of a concept is salient. In the next section, I will therefore go through three empirical studies to try to make the point that we have been wrong in neglecting salient semantics.

Before proceeding, it is important to address a further theoretical question: Should the study of salient features be classified within the domain of semantics? Using the term ‘salient semantics’ indeed marks a departure from traditional truth-conditional semantics. Nonetheless, there are several compelling reasons why ‘salient semantics’ is an appropriate term. First, this approach resonates with Putnam’s (1970) suggestion that prototype structures are an integral part of a term’s meaning, even though they don’t directly determine the word’s reference. Second, the exploration of salient features is fundamentally different from pragmatic analysis. While pragmatics deals with the use of language in context and the implications of that use, the study of salient features focuses on the inherent characteristics of concepts as they are understood independently of specific contexts. Semantic feature production tasks typically involve collecting features of concepts in a context-independent manner, i.e., participants are asked to name features of concepts without having first read a vignette, or having been primed about a specific subject. Third, semantics is fundamentally concerned with the meaning and interpretation of words and phrases in language. Salient features of concepts play a crucial role in how we understand and ascribe meaning to various terms and concepts. By examining these features, we gain insights into how meanings are constructed, interpreted, and conveyed in language. Or so I hope to show in the next section.

## Empirical studies

In this section of this paper, I aim to demonstrate how an analysis of the salient features of concepts enables us to achieve three key objectives: (a) pinpoint the features that are crucial in our reasoning about and with concepts, (b) scrutinize socially pertinent stereotypes, and (c) explore the intrinsic structure of concepts. To illustrate these points, I will engage in detailed discussions of three recent empirical studies, each focusing on a different concept: conspiracy theory, female/male professor, and life. Through these case studies, we will see how an investigation into the salient features not only enriches our understanding of these specific concepts but also offers broader insights into the dynamics of conceptual analysis.

### Study 1: When salience trumps necessity

There are many exciting philosophical projects which aim at revealing the necessary features of philosophically interesting concepts. My aim is not to undermine these efforts, which are integral to mainstream analytic philosophy. My overall point is different: understanding the salient features of concepts is crucial, as it enables us to address several philosophically important questions. Therefore, research that aims to identify necessary features and research targeting salient features should not only coexist but also enrich each other. That said, there are concepts, where it seems that salience trumps necessity. Let’s take a look at such a concept.

#### The case of conspiracy theory

The prevailing view among analytic philosophers is that conspiracy theories are fundamentally theories about conspiracies. This viewpoint is supported by a range of scholars, including Basham and Dentith (2016), Cassam (2019), Coady (2003), Cohnitz (2018), Feldman (2011), Harris (2018), Keeley (1999), Pigden (2007), and Räikkä (2018), although it is important to note that these scholars don’t necessarily agree on a singular definition of ‘conspiracy theory’. According to this dominant view, conspiracy theory is not a negative evaluative concept but rather seen as a descriptive concept. This implies that features like <deficient>, <crazy>, or simply <bad> are not necessary features of conspiracy theory. While some philosophers recognize that conspiracy theories are often perceived negatively, such evaluative aspects are considered to be at most pragmatic features (Pigden, 2007).

One argument for proposing a descriptive account of conspiracy theory is straightforward: by applying the method of cases, philosophers can illustrate that certain theories are identified as conspiracy theories without evaluating them negatively. Take, for instance, the theories surrounding the Watergate scandal. These are seemingly aptly categorized as conspiracy theories, given Nixon’s involvement in the conspiracy and subsequent cover-up. Yet, such theories are not regarded as epistemically flawed, irrational, or morally reprehensible. Hence, it’s reasonable to consider the Watergate scandal as a compelling counterexample to the notion that the term ‘conspiracy theory’ is inherently evaluative.^10^

Even though this putative counterexample challenges the idea that <being deficient> or a similar evaluative aspect is intrinsic to the concept conspiracy theory, it is still intriguing to explore how salient negative evaluations factor into our understanding of conspiracy theories. In this context, Napolitano and Reuter (2023) conducted a study where they gathered people’s responses using a modified semantic feature production task. Their findings indicate that negative evaluative elements are indeed salient in our representation of conspiracy theories. However, their approach diverged from the standard methodology; they asked participants to identify features they deemed necessary for a concept to qualify as a conspiracy theory. To build on this, I implemented another semantic feature production task, with a straightforward prompt: “Please tell us: Which features are characteristic of a conspiracy theory?” In this task, 40 participants were given three fields to input three features, aiming to gain further insight into the salient features of conspiracy theory.

In this study, one participant listed only examples of conspiracy theories, such as ‘flat earth’, ‘hollow earth’, and ‘lizard’, rather than identifying features. Among the remaining 39 participants, 34 participants (which constitutes 87% of the sample) included at least one negative evaluative term in their response. These terms ranged from ‘ambiguous evidence’ to descriptors like ‘far-fetched’, ‘confusing’, ‘misleading’, ‘outlandish’, ‘self-importance’, ‘gossip’, ‘arrogance’, and ‘lies’, indicating that negative evaluation is a highly salient aspect in how people conceive of conspiracy theories. The responses of the first 15 participants, as shown in Table 1, further illustrate this trend, providing a detailed view of how these theories are commonly characterized.^11^

**Table 1:** Responses of the first 15 participants in the semantic feature production task

Participant	1st Term	2nd Term	3rd Term
Person 1	Hopefulness	Arrogance	Over confidence
Person 2	Lies	Assumptions	Belief
Person 3	Unlikely outcomes	Unable to pinpoint sources	Sensationalism
Person 4	Not educated	Paranoid	Poor
Person 5	Unlikely	Far-fetched	Unbelievable
Person 7	Paranoia	Theories	Falsehoods
Person 8	Little evidence	Somewhat mysterious	Extreme ideas
Person 9	Technology	Government involvement	Allegation of suppression of info
Person 10	Lies	Fear	Distrust
Person 11	Made up	Sensational	Far fetched
Person 12	Sinister Motivates	Going against expert analysis	Paranoia
Person 13	Wild thoughts	Terrible sources	Gullible people
Person 14	Paranoia	Suspicion	Deception
Person 15	Usually false	Outlandish	The government are behind everything

#### Discussion

The results of the semantic feature production task on conspiracy theory demonstrate that an overwhelming majority think that negative features like <far-fetched> and <outlandish> are characteristic features of conspiracy theory. Thus, taking semantic feature production tasks to be in the business of revealing salient features, a highly salient feature in our representation of conspiracy theories is something akin to <is epistemically deficient>. However, let us not forget, that the concept conspiracy theory does not seem to necessarily be an evaluatively negative concept. The case of the Watergate scandal provides an intuitively compelling example that conspiracy theories can be true, justified and rational theories. So, what shall we do with our findings?

To truly understand and interpret people’s attitudes towards conspiracy theories, it is important to grasp the salient features of conspiracy theory. Similarly, comprehending how individuals reason with the term ‘conspiracy theory’ requires an understanding of these salient features. For the purpose of conceptual engineering, particularly in relation to everyday understanding of ‘conspiracy theory’, identifying these salient features is crucial. Simply analyzing specific instances, such as the Watergate scandal, falls short in providing the necessary insights for these explorations. Consequently, being aware of the necessary features required to apply the term ‘conspiracy theory’ has limited utility in offering constructive responses.

### Study 2: On stereotypes

Exploring salient features not only provides insights into how people reason and understand various concepts, but it is also crucial for understanding how certain stereotypes take shape. Both psychological and philosophical research extensively examine aspects such as (a) identifying prevalent stereotypes and biases, (b) tracing their origins, (c) analyzing their ethical implications, and (d) exploring potential interventions. Philosophers, in particular, are adept at addressing the ethical consequences of stereotypes and biases within the context of broader societal injustices and specific instances of discrimination. Additionally, philosophers across various disciplines are trained to critically assess the vehicles of thought, i.e., the concepts and language we employ to describe and think about the world. While modifying the language and concepts we use might not always be the most direct or effective method to combat harmful stereotypes, the prevailing consensus is increasingly acknowledging that our words and concepts are not neutral and require careful consideration and, potentially, change.

Stereotypical thinking often arises from the salient features of the concepts we hold, a phenomenon also highlighted in the research by Fischer and Engelhardt (2020). It is common for these stereotypes to manifest in beliefs* such as Asians excelling in mathematics or male Italians being sexist. However, these beliefs, as indicated by the asterisk, need not be explicitly endorsed; they are often held implicitly, as discussed by Holroyd et al. (2017) and Schwitzgebel (2010). Importantly, these stereotypical features are not viewed as necessary for the application of a concept. For instance, no one genuinely believes that being proficient in math is a necessary characteristic of the concept Asian person. Rather, such stereotypes reflect the traits that are conceived of as salient within certain groups, like Asians or male Italians. These conceptions highlight how stereotypes inform our understanding of different social groups. The study presented here emphasizes the critical importance of examining salient features in discussions surrounding stereotypes.

#### The case of female and male professor

In an influential article, Leslie et al. (2015) show that women are under-represented in fields were brilliance is believed to be a more important determinant of success than hard work. They also provide an explanation for this finding. They hypothesize that “women are stereotyped as not possessing such talent.” (2015, p. 262). This hypothesis would indeed explain the empirical finding that women are under-represented in fields such as mathematics, physics, etc.

Surprisingly, despite the availability of experimental methods, Leslie et al.’s hypothesis hasn’t been directly tested. To address this, Del Pinal et al. (2017) conducted a semantic feature production task to identify the most salient features associated with the concepts of female professor and male professor. If Leslie and colleagues’ assertion is accurate—that women are stereotypically believed to lack brilliance—then this belief should be reflected in the salient features identified for female professor. This approach offers a direct empirical test of the hypothesis, seeking evidence for the stereotype in question.

A total of 312 participants were recruited via Amazon Mechanical Turk. Participants were requested to write down features for specific social categories. They were randomly assigned either to one of the two test conditions (female professor or male professor), or to one of four control conditions (female baker, male baker, female actress, female actor). The target stimuli were as follows:

Imagine that Mary/Jack is a professor at a university [an actor/actress; a baker].

Please list five features that you think are typical of Mary/Jack.

(from Del Pinal et al., 2017)

**Table 2:** Results of the semantic feature production task in Del Pinal et al. (2017)

	Male (%)	Female (%)	$\chi ^2$	p
Professor + hardworking	21.3	39.5	4.71	0.030
Professor + smart	72.4	76.6	1.26	0.473
Actor/actress + hardworking	7.5	10.9	0.36	0.547
Actor/actress + smart	17.0	10.9	0.77	0.380
Baker + hardworking	24.5	19.2	0.41	0.522
Baker + smart	10.2	1.9	3.09	0.079

The results are displayed in Table 2 and reveal two noteworthy findings: First, contrary to the hypothesis that female professors are stereotypically viewed as less brilliant, the participants in this semantic feature production task attributed terms such as ‘smart’ and ‘intelligent’ equally to both female and male professors. This finding provides little support for the explanation proposed by Leslie and colleagues regarding the stereotype of female professors’ brilliance. Second, although there was no significant difference in the frequency of terms like ‘smart’ and ‘intelligent’ being associated with both genders, a notable difference emerged in the proportion of participants who used terms synonymous with ‘hardworking’. Intriguingly, the term ‘hardworking’ and its synonyms were almost twice as likely to be associated with female professors compared to male professors.

In a subsequent study, Del Pinal and colleagues offered an empirically supported alternative explanation for the perceived association between gender and brilliance. Their study focused on exploring gender-specific associations between being ‘smart’ and ‘hardworking’. Participants were asked to estimate the number of hours per week a female and a male professor would need to work. The findings were telling: when the smartness of female professors was emphasized, they were perceived as needing to work more hours compared to the control condition. In contrast, this increase in perceived work hours was not observed for male professors when their smartness was highlighted. Overall, the research by Del Pinal et al. suggests that the stereotype linking brilliance and gender exists within the dependency networks of the concepts female professor and male professor, but this stereotype is not evident at the level of feature salience.

#### Discussion

Semantic feature production tasks not only enable the identification of a concept’s most salient features (as demonstrated in Study 1), they also provide insights into the extent and nature of stereotypical thought. As such, these tasks offer a relatively efficient and direct method for examining various hypotheses related to the stereotypes held by people. In the specific instance of Del Pinal et al.’s study, this approach was instrumental in casting doubt on Leslie’s hypothesis that women are stereotypically perceived as having less intelligence.

### Study 3: On the structure of concepts

In the previous two subsections, we have seen that detecting the salient features of concepts allows us (a) to identify those features that are likely to dominate people’s thinking in various cognitive processes, and (b) to examine those salient features that are likely to play a crucial role in stereotypical thinking. In the following study (Study 3), I aim to show that investigating salient features through semantic feature production tasks can provide us with insights not only into the meaning of concepts but also into the structure of concepts.

#### The case of LIFE

The concept life has been highlighted to be particularly resistant to a (classical) definition that receives widespread agreement. Chyba and McDonald (1995, p. 216), for example, claim that “it is now a commonplace that the various proposed definitions [of life] virtually all fail”. While Chyba and McDonald might be right in their assessment, they do not provide an answer of why that is the case. In trying to make sense of the state of confusion, drawing on Wittgenstein’s notion of family resemblance, Pennock (2012, p. 5) claims that “life is a cluster concept with fuzzy boundaries”. However, conclusions in regards to the structure of the concept life seem to be at best premature without any empirical evidence on the matter.

Beisbart and Reuter (2021) presented laypeople with a semantic feature production task. More specifically, they asked 102 participants (65 female, 36 male, 1 non-identied), to write down up to three answers to one of the following two different versions of a semantic feature production task:

- First version. “Which features are characteristic of species of living beings? You can name up to three features.”
- Second version. “Which features do you think distinguish species of living beings from non-living entities?”

The answers of the first 15 participants are displayed in Table 3. The most frequently named responses fell into the categories <growth> (47%), <breathing> (46%), <reproduction> (35%) and <nutrition> (31%). People gave responses indicative of what “living beings do at the level of a whole living being, and most of these features are observable for many life forms” (2021). People hardly gave answers that were classified to refer to the material (organic matter) or structure of the underlying material (cells (10%)).

**Table 3:**
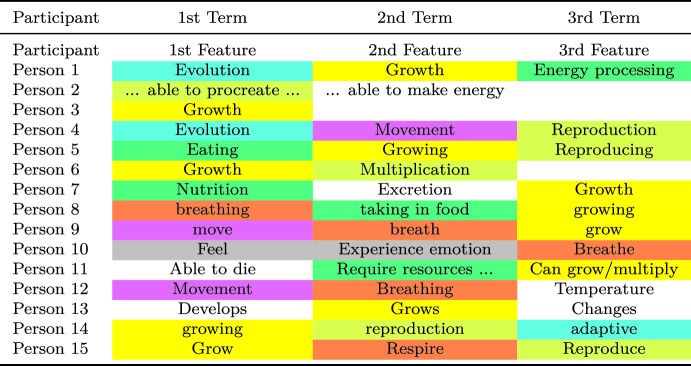
Responses of the first 15 participants to the semantic feature task

Beisbart and Reuter used eight categories to classify the responses: breathing cells (no response among the first 15 participants), evolution, growth, movement, nutrition, perception/consciousness, reproduction. The cells left white indicate responses that were not categorized. Table taken from Beisbart and Reuter (2022)

In a second study, Beisbart and Reuter aimed to find out which features of living beings people consider to be universal, i.e., which features are thought to hold for all species of life. In order to examine which features people consider to be universal, they asked them “What percentage of species consist of living beings that [feature]?” People’s answers were measured on a scale ranging from 0 to 100% in steps of 1%. In contrast to the semantic feature production task, an entirely different outcome emerged. Whereas only 10% of the participants named <cells> or <material> in the semantic feature production task, a whopping 68% thought that 100% of species of living beings are made of organic material, and 64% considered all species to be made of cells. In contrast, <growth> and <nutrition> received much lower numbers.

#### Discussion

The research conducted by Beisbart and Reuter indicates that focusing solely on necessary features overlooks critical elements of the concept of life. A key finding from their empirical studies is the distinction between salient features on the one hand, and universal features on the other. This raises the question: How can we interpret these differences? Beisbart and Reuter suggest that this division mirrors the inherent structure of the concept of life, which they believe is a natural kind concept embodying both an essence and observable surface properties. Their proposal outlines three aspects: First, they posit that life is conceived of as a natural kind, underpinned by an esseence, such as cellular or organic composition. Second, they argue that people identify this natural kind through salient macroscopic features, like <growth> and <nutrition uptake>. Third, they contend that the essence can be identified by current scientific knowledge.

Claims, according to which life cannot be defined (Machery, 2012), or that life is a family resemblance concept (Pennock, 2012) were not based on empirical data that took into account the importance of salient features. However, this case shows that finding out about the structure of a concept often requires investigating the salient features of a concept. The results of these investigations can then help us to capture more precisely the semantics of our concepts as well as help developing more reliable and empirically grounded theories.

## General discussion

The identification of the salient features of concepts plays at most a minor role in philosophical studies so far. Whenever philosophers are concerned with analyzing a certain concept, they are likely to focus on what the necessary and jointly sufficient features of a concept are. Experimental philosophers have certainly departed from the obsession on necessary features in many philosophical projects. However, even experimental philosophers seem to be strongly focusing on the traditional program of uncovering necessary and sufficient conditions.

Psychologists have uncovered a range of cognitive processes where the salience of features plays a more crucial role than their necessity for the application of a concept. These processes encompass memory, recognition, categorization, and reasoning, among others. While psychologists have given considerable attention to the salience of features in concepts within their field, there’s a noticeable lack of focus on features of concepts that hold philosophical significance. This oversight is understandable, as concepts deemed important in philosophy may not always align with those considered relevant in psychological studies. Therefore, the divergence in interest between these two disciplines, particularly regarding concept features, is not surprising.

Despite the importance of salience for various cognitive processes, philosophers might still be justified in disregarding research into the salient features of concepts. Why is that? If our philosophical interests are only peripherally, if at all, affected by the role that salient features play, then research time and mental effort are better put into other projects. It seems, for example, that questions about how the salience of features of concepts such as free will influence how those concepts are retrieved from memory, are philosophically not overly exciting. The primary aim of this paper was therefore to make a compelling case that philosophers should be interested in salient semantics.

In the empirical part of this paper, I have presented studies that underline the importance of salient semantics for the concepts conspiracy theory, female professor, and life.^12^ It seems to me that the results of the semantic feature production tasks are indeed relevant for a range of philosophical debates, both at the level of those individual concepts as well as at the level of more general questions, like, how is evaluative content encoded in our concepts?, how do stereotypes work?, how are our concepts structured?, etc. That said, there are a lot of issues and questions one can raise about the role of the semantic feature production task. We still know relatively little about the cognitive processes involved when retrieving features of abstract concepts in a semantic feature production task. As most philosophically relevant concepts are fairly abstract, it might well be that we cannot make reliable inferences from the results of studies on concrete concepts (e.g., McRae et al., 2005; Vinson et al., 2008; Buchanan, 2019) to abstract concepts. Perhaps most worryingly, the lack of knowledge about the cognitive processes involved in semantic feature production tasks for abstract concepts, raise the very real possibility that the experiments tap into different properties of these concepts.^13^

I would like to close by briefly considering the relevance of salient semantics for two of the most central research methods in philosophy, conceptual engineering and conceptual analysis.

### Conceptual engineering

Conceptual Engineering has emerged as an umbrella term for explication and amelioration. While explication projects aim to improve our concepts to make them more fruitful for scientific purposes, ameliorative projects aim to improve our concepts (a) for better public discourse and reasoning, (b) to eliminate sources of misunderstanding and confusion, and (c) to reduce discrimination. I am certainly not the first to argue that amelioration shouldn’t rely too much on a classical conception of concepts. Other researchers (Machery, 2017: chap. 7; Fischer, 2020; Isaac, 2021a, b) have highlighted the need for more “psychological” approaches both for identifying which aspects of our concepts need improvement, and also for determining how new concepts can be more successfully implemented. The results of the semantic feature production tasks for conspiracy theory and female professor reinforce this claim. Without knowing how salient the evaluative content of conspiracy theory is, we do not know, for example, the degree to which people may talk past each other when discussing conspiracy theories, the degree to which the use of the term ‘conspiracy theory’ has the potential to disparage certain theories and advocates of those theories, etc. Furthermore, semantic feature production tasks are likely to allow insights into how new or redefined concepts inherit unwanted features from related concepts. Thus, when it comes to conceptual engineering, the need for salient semantics seems to be immanently plausible.

### Conceptual analysis

Conceptual analysis is traditionally conceived to be the process of (i) providing sets of necessary and jointly sufficient features of concepts. Successful analyses are often taken to be (ii) referentially invariant and (iii) feasibly performed by individual reflection on cases. There is a sense in which salient semantics has no bearing whatsoever on conceptual analysis, given its focus on the necessity of features. That said, conceptual analysis, as traditionally conceived, has received severe criticism. One strand of criticism takes the underlying assumption of the classical theory of concepts to be misguided (Chalmers & Jackson, 2001). A second strand takes experimental–philosophical studies to show huge variation in the reference of concepts between people on an individual level, as well as groups of people on a cultural level (Machery et al., 2017; Reuter and Sytsma 2020; Weinberg et al. 2001). A third strand looks for additional methods to circumvent various problems with the method of cases, e.g., corpus-analytic approaches (Andow, 2015; Fischer et al., 2015; Hansen et al., 2021; Reuter, 2011; Sytsma et al., 2019). As a consequence of these objections, we find that many philosophers entertain a looser concept of conceptual analysis that is not tied to the classical theory of concepts, not tied to referentially invariant concepts, and not tied to the method of cases. Although nowadays many philosophers don’t do conceptual analysis as traditionally conceived, they, of course, still analyze concepts. Once we adopt a wider and more liberal perspective on what it means to analyze concepts, there is no good reason to exclude the investigation of salient features of concepts from the philosophical task of analyzing concepts.

## Conclusion

The primary objective of this paper was to emphasize the significance of researching salient features of concepts. To underscore this point, I have detailed three case studies, each exemplifying a unique context in which understanding salient features is not just philosophically intriguing, but also essential for advancing knowledge in specific areas. First, the case of conspiracy theory shows the importance of identifying salient features, which are pivotal in shaping our understanding and reasoning about conspiracy theories. Second, examining the concept of female/male professor highlights how salient features are instrumental in analyzing socially relevant stereotypes. Finally, the study of life demonstrates the need of pinpointing salient features to uncover the structure of concepts. While these three examples provide a glimpse into the substantial philosophical utility of salient semantics, they represent just a fraction of its potential applications and impacts.

## Footnotes

1 In this paper I use the term ‘features’ to denote components of concepts, whereas the term ‘properties’ is reserved for aspects of objects. The term ‘attributes’ is used to refer to the logical status of features, e.g., necessary, sufficient, typical, salient.

2 Other attributes of features like *diagnostic* *central*, and *sufficient* do not play an important role for the purposes of this paper.

3 Sometimes, especially in discussions on natural kinds, the theory–theory of concepts is considered as an alternative to the classical theory of concepts. However, even then, the essential features of a natural kind concept are the ones that interest philosophers most, and not so much the superficial features that might be highly salient.

4 For a discussion on the significance of the classical theory of concepts in philosophy, see especially Laurence and Margolis (1999).

5 Other methods like the cancellation paradigm (Willemsen & Reuter, 2021) corpus analysis (e.g. Hansen et al., 2021; Sytsma et al., 2019), eye tracking (e.g., Fischer et al., 2021), and statistical learning (Nichols, 2021) have entered the experimental–philosophical stage, which are usually not utilized to test for necessary features of concepts.

6 Recently, the philosophical study of salience has received some attention (see e.g., Archer, 2022; Michaelson & Nowak, 2022; Ratcliffe & Broome, 2022; Watzl, 2022; Whiteley, 2022). However, note that while these philosophers, as well as many psychologists and linguists, are focused on the salience of a *stimulus* that can capture people’s attention (Wu, 2011), e.g., a red fire hydrant on a sidewalk, this paper is focused on the salience of features of a concept (Breheny, 2008; Fischer & Sytsma, 2021; Fischer et al., 2021).

7 Rutschmann and Wiegmann state that “[o]ur results indicate that the intention to deceive is not a necessary condition for lying.” (2017, p. 438). Turri and Turri claim that “[o]ur main conclusion is that, contrary to the standard view, falsity is a necessary component of lying and, thus, that lying has an important non-psychological element.” (2015, p. 162) and Wiegmann and Willemsen hold that “[i]n this paper, we showed that for something to be a lie, subjective falsity at the semantic level is not necessary.” (2017, p. 3521)

8 Other methods that have been used to identify salient features of concepts are sentence completion tasks (e.g., Fischer & Engelhardt, 2020) as well as non-literal comparisons, see e.g., Barsalou (1982) and Ortony et al. (1985).

9 It might be argued that the example of a duck that was given to participants biased people into giving responses that are typical features of concepts. However, similar outcomes have been observed in other methodologies (see e.g., Hampton, 1979), and the results presented in this paper suggest that the example was not biasing people into giving typical features.

10 Some of Napolitano and Reuter (2023)’s results put pressure on the idea that many people would consider the Watergate scandal a conspiracy theory.

11 Through their corpus-analytic studies, Napolitano and Reuter (2023) and Reuter and Baumgartner (2024, forthcoming) have provided evidence that the term ‘conspiracy theory’ is commonly used with negative implications. This research leads to an intriguing and largely unexplored question: How effectively can corpus analysis uncover the salient features of a concept? As it stands, the extent to which this method can yield comprehensive insights into concept characterization remains an open area for investigation.

12 For further and very recent applications of the semantic feature production task for philosophical projects, see Reuter (2019) on the concept of colleague Erbach et al. (2023)’s work on race, as well as Reuter, Latham and Varga’s (ms) research on the concept of health.

13 I owe these criticisms in part to Matteo Colombo (personal communication).
